# Oesophageal heat exchangers with a diameter of 11mm or 14.7mm are equally effective and safe for targeted temperature management

**DOI:** 10.1371/journal.pone.0173229

**Published:** 2017-03-14

**Authors:** Daniel C. Schroeder, Maria Guschlbauer, Alexandra C. Maul, Daniel A. Cremer, Ingrid Becker, David de la Puente Bethencourt, Peter Paal, Stephan A. Padosch, Wolfgang A. Wetsch, Thorsten Annecke, Bernd W. Böttiger, Anja Sterner-Kock, Holger Herff

**Affiliations:** 1 Department of Anaesthesiology and Intensive Care Medicine, University Hospital of Cologne, Kerpener Straße 62, Cologne, Germany; 2 Department of Experimental Medicine, University Hospital of Cologne, Cologne, Germany; 3 Institute of Medical Statistics, Informatics and Epidemiology, University Hospital of Cologne, Kerpener Straße 62, Cologne, Germany; 4 Barts Heart Centre, St Bartholomew's Hospital, Barts Health NHS Trust. Queen Mary University of London, London, United Kingdom; 5 Barmherzige Brüder Salzburg Hospital, Department of Anaesthesiology and Critical Care Medicine, Kajetanerplatz 2, Salzburg, Austria; University of Illinois at Urbana-Champaign, UNITED STATES

## Abstract

**Background:**

Targeted temperature management (TTM) is widely used in critical care settings for conditions including hepatic encephalopathy, hypoxic ischemic encephalopathy, meningitis, myocardial infarction, paediatric cardiac arrest, spinal cord injury, traumatic brain injury, ischemic stroke and sepsis. Furthermore, TTM is a key treatment for patients after out-of-hospital cardiac-arrest (OHCA). However, the optimal cooling method, which is quick, safe and cost-effective still remains controversial. Since the oesophagus is adjacent to heart and aorta, fast heat-convection to the central blood-stream could be achieved with a minimally invasive oesophageal heat exchanger (OHE). To date, the optimal diameter of an OHE is still unknown. While larger diameters may cause thermal- or pressure-related tissue damage after long-term exposure to the oesophageal wall, smaller diameter (e.g., gastric tubes, up to 11mm) may not provide effective cooling rates. Thus, the objective of the study was to compare OHE-diameters of 11mm (OHE11) and 14.7mm (OHE14.7) and their effects on tissue and cooling capability.

**Methods:**

Pigs were randomized to OHE11 (N = 8) or OHE14.7 (N = 8). After cooling, pigs were maintained at 33°C for 1 hour. After 10h rewarming, oesophagi were analyzed by means of histopathology. The oesophagus of four animals from a separate study that underwent exactly the identical preparation and cooling protocol described above but received a maintenance period of 24h were used as histopathological controls.

**Results:**

Mean cooling rates were 2.8±0.4°C°C/h (OHE11) and 3.0±0.3°C °C/h (OHE14.7; p = 0.20). Occasional mild acute inflammatory transepithelial infiltrates were found in the cranial segment of the oesophagus in all groups including controls. Deviations from target temperature were 0.1±0.4°C (OHE11) and 0±0.1°C (OHE14.7; p = 0.91). Rewarming rates were 0.19±0.07°C °C/h (OHE11) and 0.20±0.05°C °C/h (OHE14.7; p = 0.75).

**Conclusions:**

OHE with diameters of 11 mm and 14.7 mm achieve effective cooling rates for TTM and did not cause any relevant oesophageal tissue damage. Both OHE demonstrated acceptable deviations from target temperature and allowed for an intended rewarming rate (0.25°C/h).

## Introduction

Today, targeted temperature management (TTM) is widely used in critical care settings for conditions including hepatic encephalopathy [1], hypoxic ischemic encephalopathy [2], meningitis [3], myocardial infarction [4], paediatric cardiac arrest [5], spinal cord injury [6], traumatic brain injury [7], ischemic stroke [8] and sepsis [9]. Particularly, TTM is a key therapy after out-of-hospital cardiac-arrest (OHCA) [10]. Invasive intravascular cooling devices (IVD) that facilitate cooling directly via the blood stream are commonly used for TTM. IVD are effective and reliable for maintaining a stable pulmonary target temperature [11]; their insertion, however, is time intensive [12] and may be associated with adverse events including bleeding and thrombosis [12].

Since the oesophagus is situated next to heart, aorta and inferior vena cava the oesophagus may be suitable for heat convection to the central body during TTM [13, 14]. Recently, effective cooling rates could be achieved using a minimally invasive oesophageal heat exchanger (OHE), which can be inserted easily, quickly and safely [15–17]. In addition to that, adherence to the target temperature during maintenance and rewarming period was accurate, which is important to avoid e.g. overcooling and adverse events such as electrolyte disorders or shivering [12]. To date, the optimal diameter of an OHE is still unknown. While larger diameters comparable to a Sengstaken-Blakemore tube (36mm) may cause thermal- or pressure-related tissue damage after long-term exposure to the oesophageal wall, smaller diameter (e.g. gastric tubes, up to 11mm) may not provide effective cooling rates. Thus, the objective of the study was to compare two specific OHE-diameters and their effects on tissue and cooling capability.

## Methods

### Animal experiments

This project was approved by the local animal care committee and governmental authorities (Landesamt für Natur-, Umwelt- und Verbraucherschutz NRW; 84–02.04.2014.A081; 84–02.04.2014.A157). All procedures were in accordance with the German Laws for Animal Protection. Animal care and use was performed by qualified physicians, supervised by a veterinarian. The study protocol complies with the Animals in Research: Reporting In Vivo Experiments (ARRIVE) guidelines for reporting in vivo experiments in animal research [18].

### Perioperative management and anaesthesia

This study was performed using 23 healthy, 8–10 week–old female pigs (Landrace x Pietrain). Weight of the pigs was 27.2±1.2kg (OHE11), and 29.8±2.6kg (OHE14.7; p = 0.03). Six animals served as pilots. Straw bedded pens (9.3 m^2^) were provided, ambient temperature was 20°C. Animals were fasted overnight before the experiment. Animals received an intramuscular injection of azaperone (2 mg/kg; Stresnil, Janssen, Neuss, Germany), ketamine (20 mg/kg; Ketavet 100, Pfizer, Berlin, Germany) and atropine (0.02 mg/kg, Braun, Melsungen, Germany) for premedication. After transporting the animals to the operating theatre, a 20 gauge catheter (Vasovet, Braun, Melsungen, Germany) was placed in the lateral auricular vein. Animals were placed in a supine position, propofol was administered (2 mg/kg, Fresenius, Bad Homburg, Germany), and the trachea was intubated with a 6.0–6.5 mm endotracheal-tube (Teleflex Medical, Kernen, Germany). Thereafter, pigs were ventilated with pressure-control (Fabius GS, Dräger, Lübeck, Germany) and 30% oxygen, 14 breaths/min. Tidal volume was adjusted to maintain normocapnia (PaCO_2_ 35-45mmHg). PaO_2_ was maintained >70mmHg. Anaesthesia was maintained with propofol (5–7 mg/kg/h), midazolam (1.2 mg/kg/h; Rotexmedica, Trittau, Germany) and fentanyl (12–15 mg/kg/h; Fentanyl, Rotexmedica, Trittau, Germany). Lactated Ringer´s solution (5–10 ml/kg/h, Fresenius Kabi, Bad Homburg, Germany) was administered [19]. A standard lead II electrocardiogram was used to monitor cardiac rhythm (Philips Medizinsysteme, Böblingen, Germany). If cardiovascular variables indicated a reduced depth of anaesthesia, additional propofol, midazolam or fentanyl was given. Each animal received enrofloxacine (2.5 mg/kg i.m.; Baytril; Bayer, Leverkusen, Germany) after induction of anaesthesia.

With arterial glucose <3.5 mmol/l 250ml glucose (Glucosteril 5%; Fresenius Kabi; Bad Homburg, Germany) were administered. If mean arterial blood pressure decreased to <50mmHg norepinephrine (0.1μg/kg/min i.v.; Arterenol, Sanofi-Aventis, Frankfurt am Main, Germany) was supplemented.

### Surgical preparations

A 7F saline-filled catheter (Arterial leadercath, Vygon, Ecouen, France) was advanced via femoral cut-down into the femoral artery to sample arterial blood and measure blood pressure (Philips M1097A, Philips Medizinsysteme, Böblingen, Germany). A 14G catheter (Arrow International, Reading, USA) was advanced into the femoral vein for continuous drug application. A 5F pulmonary artery catheter (Arrow International, Reading, USA) was placed via cut-down in the internal jugular vein and subsequently advanced into the pulmonary artery to measure the pulmonary artery temperature. A 12Ch catheter (Balloon Catheter, Teleflex Medical, Kernen, Germany) was inserted into the bladder to drain urine. Blood gases were measured according to a predefined protocol (ABLFlex800, Radiometer, Willich, Germany). A manufactured temperature probe using a PT 100 (e.g. P-M-A-6-100-0-TS-2, Omega Engineering GmbH, Deckenpfronn, Germany) was placed in the operating theatre to measure ambient temperature.

### Experimental TTM-study-protocol

**Fig 1:**
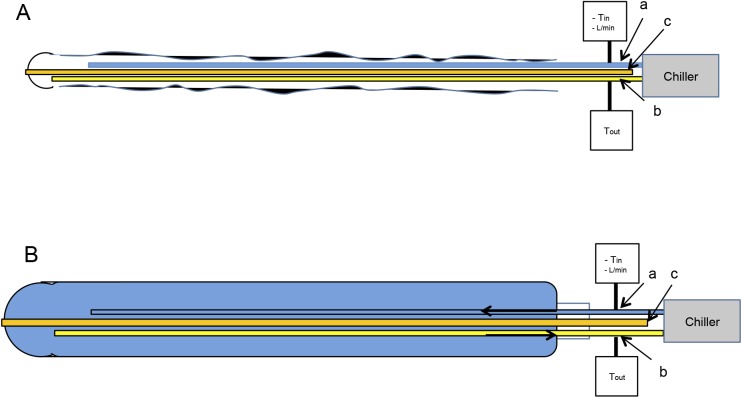
Design of Oesophageal Heat Exchanger. The OHE consisted of silicone designated for medical use. The tube (500mm length) consisted of three integrated tubes: (a) the outlet tube supplied water from the temperature regulating device (HICO Variotherm 555, Hirtz & Co.KG, Cologne, Germany), and was connected to the inlet tube (b), which withdrew the water back to the chiller. A third tube (c) provided gastric suctioning. Purified water served as temperature regulating agent. Water temperature was assessed at the inlet (*T*_*in*_) and the outlet (*T*_*out*_) of the OHE. Water could be cooled down to a minimum of 3°C or warmed to a maximum of 41°C. With a feedback loop, which registered the pulmonary artery temperature (Gold standard), the water temperature was continuously adjusted to the requirements of the study protocol. Water flow rate (*L*/min) was measured in the forward line. Both OHE11 and OHE14.7 were inserted in uninflated (A) conditions to protect the oesophageal epithelium from desquamation and avoid unnecessary contact pressure. Immediately after initiation of cooling, OHE deflated (B) to their particular diameters. Under clinical circumstances, a blind advance of the OHE similar to a gastric tube may conceivable.

During instrumentation pigs were kept at their physiological core body temperature (38.5–39.5°C) with an air-circulating blanket (Bairhugger, 3M, Neuss, Germany). Thereafter, pigs were randomly assigned into two groups to receive either an OHE11 (n = 8) or an OHE14.7 (n = 8). After insertion of a guide wire into the tube for gastric suctioning, the uninflated OHE was inserted into the oesophagus under laryngoscopy (27cm spatula, Karl Storz, Tuttlingen, Germany) (Fig 1). The OHE was connected to the chiller. Ambient and pulmonary artery temperatures were recorded continuously (Labview, National Instruments Germany, Munich, Germany). Haemodynamic and ventilation parameters were recorded in 15-min intervals.

At least 5 minutes before baseline measurements, air-circulating blankets were switched off and removed. Pigs were cooled with the OHE to a pulmonary artery target temperature of 33°C, maintained at 33°C for 1 hour, and then covered with a silver-blanket and rewarmed (intended rate 0.25°C/h). After 10h of rewarming, animals were euthanized with pentobarbital (80 mg/kg; Pentobarbital-Natrium, CP Pharma, Burgdorf, Germany). The oesophagus including larynx and cardia was resected and separated in four segments: laryngeal, cranial, middle and caudal segment.

### Histopathology

The oesophagus of four animals from a separate study (84–02.04.2014.A157) that underwent exactly the identical preparation and cooling protocol described above (perioperative management and anaesthesia, surgical preparations, pre-warming) but received a maintenance period of 24h and did not receive any oesophageal intervention (e.g. OHE) were used as histopathological controls. The oesophageal tissue segments were fixed in 4% formalin for 24h and embedded in paraffin. For each oesophageal tissue segment slices (2–3 μm thickness) were cut. For the control group (n = 4 animals) laryngeal, cranial, middle, and caudal segment slices were cut (n = 4 each). For OHE11 (n = 8 animals) laryngeal (n = 7), cranial (n = 8), middle (n = 8), and caudal (n = 8) segment slices were performed. For OHE14.7 (n = 8) laryngeal (n = 3), cranial (n = 8), middle (n = 8), and caudal (n = 8) slices were performed (Table 1). Slices were mounted on glass and stained with haematoxilin and eosin (HE). Light microscopy (Olympus BX40; Olympus Deutschland GmbH, Hamburg, Germany) was used, photomicrographs were taken (Olympus DP25; cellSens Standard 1.11, Olympus Deutschland GmbH, Hamburg, Germany), and examined in 20x magnification by a veterinary pathologist, blinded to the experimental setting. Tissue damage was classified according to a scoring protocol modified from Lequerica et al. who evaluated hyperthermic oesophageal tissue damage after cardiac ablation via the oesophagus [20]. The score was further developed and adapted to requirements of this study by a veterinarian pathologist. In brief, a numerical score for the following histological characteristics was assessed: epithelial hyperplasia, epithelial lesions, oedema (transmural), hyperaemia, reactive submucosal glands, intraepithelial inflammation and submucosal inflammation. Each location received a numerical score: 0 points: no specific findings, 1 point: focal tissue alteration, 2 points: multifocal tissue alterations. Intraepithelial and submucosal inflammation received 0 points for no specific findings, 1 point if <5 mononuclear inflammatory cells per field were found and 2 points if >5 mononuclear inflammatory cells per field were found. The sum was calculated for each slice. Scores 0–4 represented mild, 5–8 moderate and >9 severe oesophageal tissue damage.

**Table 1:** Levels of Oesophageal Tissue Damage.

	Control-group (N = 4)			OHE11 (N = 8)			OHE14.7 (N = 8)		
	mean±SD	*median [IQR]*	*examined slides (n)*	mean±SD	*median [IQR]*	*examined slides (n)*	mean ±SD	*median [IQR]*	*examined slides (n)*
Laryngeal segment	3.5±1.0	4 [3–4]	4	2.3±1.1	2 [1–3]	7	3.0±0	3 [3–3]	3
Cranial segment	1.8±0.5	2 [1.5–2]	4	1.3±1.0	1 [0.5–2]	8	1.5±0.5	1.5 [1–2]	8
Middle segment	1.0±0.7	1 [0.5–1.5]	4	1.3±0.7	1 [1–2]	8	1.3±1.0	1 [0.5–2]	8
Caudal segment	0.8±1.0	0.5 [0–1.5]	4	1.0±1.4	0.5 [0–1.5]	8	0.0	0 [0–0]	8

Levels of laryngeal, cranial, middle and caudal oesophageal tissue damage in an untreated control group and after application of OHE11 (N = 8) and OHE14.7 (N = 8). The number of injuries was not normally distributed and therefore is described by mean ± median [interquartile range (IQR)].

### Thermal energy (dt)

To quantify the thermal energy transferred by the OHE during the cooling period a mathematical model described by Naiman et al. was used [21]. In brief,

$${dt}_{patient}=c_{pm}(△T*-△T_{Ref})$$

*dt*_*patient*_(*W*): energy gained by the patient; *c*_*p*_: specific heat of the fluid (4.186*kJ*/[*kg* * *K*]); *m*: mass (water) flow rate (*L*/min); △T*: average temperature change in the patient (*T*_*in*_–*T*_*out*_); (△*T*_*Ref*_): average temperature change in ambient conditions. Since ambient temperature did not show fluctuations, this parameter was ignored.

### Statistical analysis

Calculations and statistical analysis were performed with SAS 9.3 (SAS Institute, Inc., Cary, NC) and IBM SPSS Statistics (version 23.0, IBM, Armonk, NY). Graphs were made using Graphpad Prism V.6.0 (Graphpad Software, San Diego, CA and IBM SPSS Statistics (version 23.0, IBM, Armonk, NY). All data are expressed as mean±standard deviation (SD). The number of injuries was not normally distributed and was therefore described by median [interquartile range (IQR)]. Due to the small sample size and distribution, group differences were tested by nonparametric methods, U-Mann-Whitney or Kruskal-Wallis test. P<0.05 was considered statistically significant.

## Results

### Animal experiments

Average ambient temperature was: Cooling period: 21.75±0.53°C (OHE11) and 22.14±0.9°C (OHE14.7; p = 0.172); maintenance: 21.61±0.68°C (OHE11) and 21.68±0.55°C (OHE14.7; p = 0.674); rewarming period: 21.01±0.56°C (OHE11) and 21.5±0.40°C (OHE14.7; p = 0.074). All OHE were inserted without complications. Entire time of placement in the oesophagus was 772.1±20min (OHE11) and 755±5.4min (OHE14.7, p = 0.09). One animal of the OHE14.7-group could not be included and needed to be euthanized after development of signs of septicaemia (HR>130; MAP<70) at 04h 15min after start of cooling. Data of the cooling period of one animal in the OHE14.7 group was excluded due to a software error.

### Cooling period

**Fig 2:**
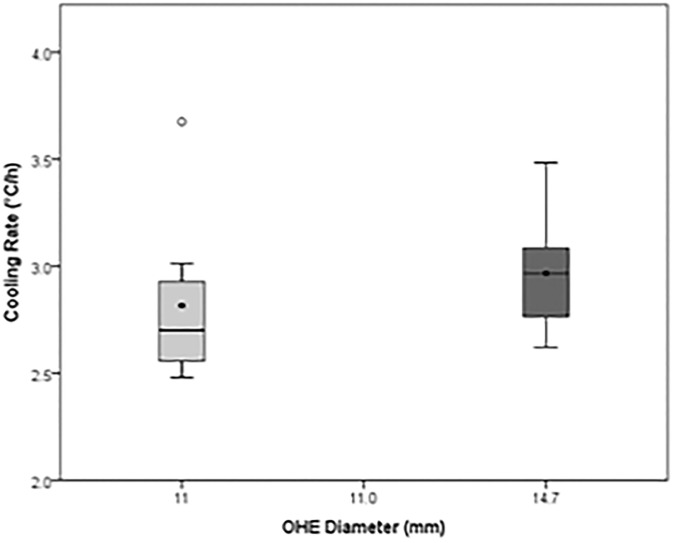
Mean cooling rates of OHE11 (N = 8) and OHE14.7 (N = 7) during cooling period.

**Fig 3:**
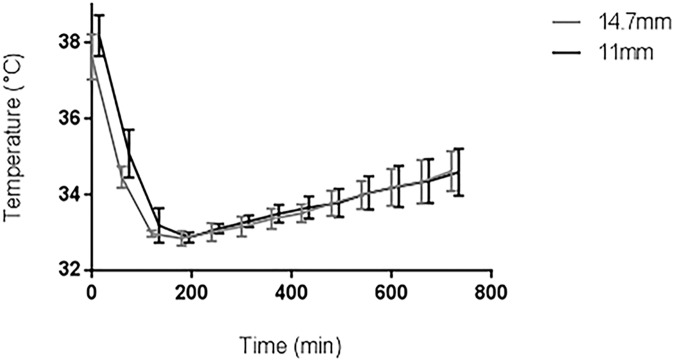
Pulmonary artery temperature profile of OHE11 (N = 8) and OHE14.7 (N = 8) during cooling period, maintenance and rewarming period.

Onset of cooling was 38.2±0.5°C (OHE11) and 37.7±0.5°C (OHE14.7). Cooling rates were 2.8±0.4°C/h (OHE11) and 3.0±0.3°C/h (OHE14.7; p = 0.20) (Figs 2 and 3). *T*_*in*_ during cooling period was 3.34±0.16°C (OHE11) and 3.42±0.48°C (OHE14.7; p = 0.563). *T*_*out*_ Tout was 5.57±0.57°C (OHE11) and 4.31±0.50°C (OHE14.7; p = 0.004). Water flow rate was 0.40±0.08 *L*/min (OHE11) and 0.91±0.05 *L*/min (OHE14.7 p = 0.001). *Q*_*patient*_ was -60,6±11.01 *w* W (OHE11) and -56.34±12.99 *w* W (OHE14.7; p = 0.418).

### Histopathology

**Fig 4:**
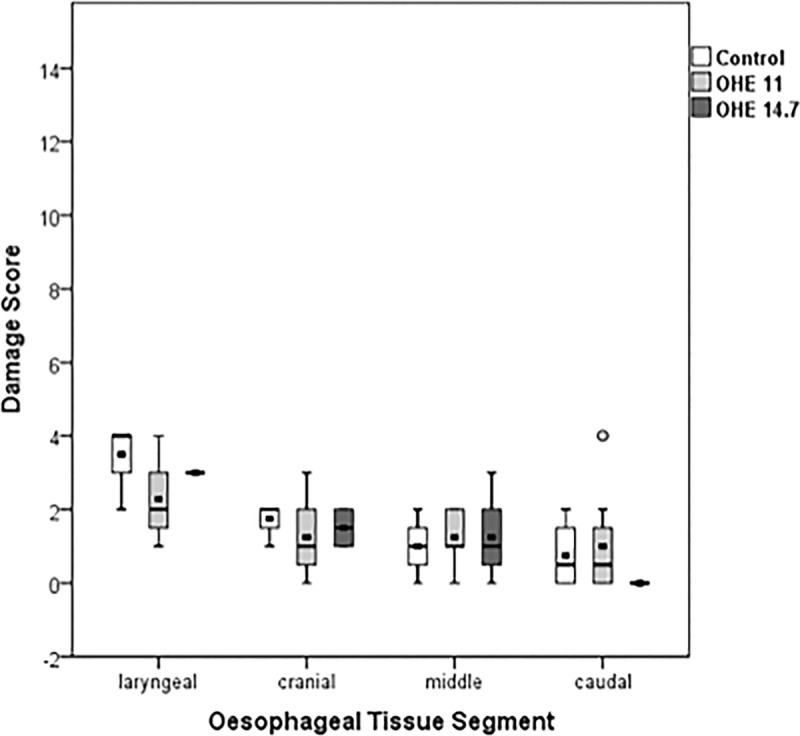
Oesophageal tissue damage was assessed for controls (N = 4), OHE11 (N = 8) and OHE14.7 (N = 8) using a modified scoring protocol according to Lequerica et al. Scores 0–4 represent a mild, scores 5–8 represent a moderate and scores >9 represent a severe oesophageal tissue damage.

**Fig 5:**
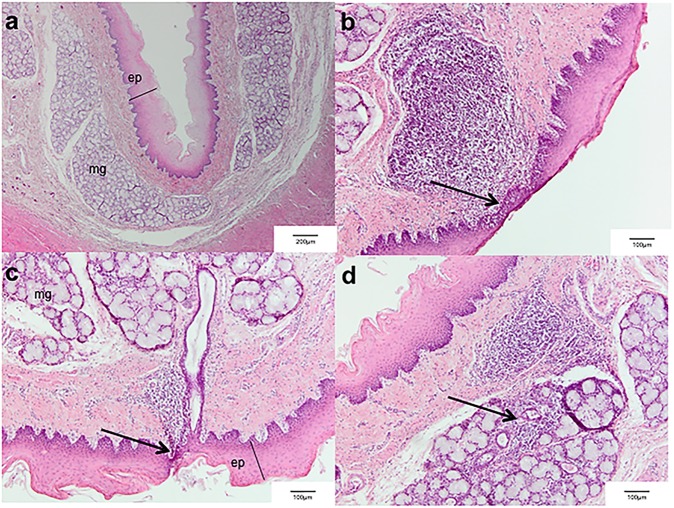
(a.) Representative undamaged cranial oesophageal tissue segment after treatment with OHE11 (ep: epithelium layer, mg: mucosal glands). No damage was found in oesophageal lamina mucosa, submucosa, muscularis, and adventitia. Epithelial layer thickness was inhomogeneous since cells of the multilayered squamous epithelium are physiologically desquamating. Scale bar: 200μm; (b/c.) Laryngeal oesophageal tissue segment after treatment with OHE11. Mononuclear inflammatory cells infiltrate the epithelial layer (arrow) of the mucosa. Activated lymphoid tissue is evident transmurally in the oesophageal wall. Scale bar: 100μm; (d.) Cranial oesophageal tissue segment after treatment with OHE14.7. Submucosal glands are infiltrated with inflammatory cellular infiltrates in the lamina submucosa (arrow). Scale bar: 100μm.

Tissue damage in the laryngeal segments resulted in a comparable score of 3.5±1.0 (control), 2.3±1.1 (OHE11) and 3.0±0 (OHE14.7). Tissue damage in the cranial segment resulted in a comparable score of 1.8±0.5 (control), 1.3±1.0 (OHE11) and 1.5±0.5 (OHE14.7). Tissue damage in the middle segment resulted in a comparable score of 1.0±0.7 (control), 1.3±0.7 (OHE11) and 1.3±1.0 (OHE14.7). Tissue damage in the caudal segment resulted in a comparable score of 0.8±1.0 (control), 1.0±1.4 (OHE11), and no tissue damage (score 0) was observed in the OHE14.7-group (Table 1). Occasional mild mononuclear acute inflammatory transepithelial infiltrates were found in laryngeal tissue segments of all groups including controls, but not in other tissue segments (Figs 4 and 5, S1 Table). The one animal euthanized at 4h 15min underwent a comprehensive autopsy. Since no signs of oesophageal damage were found a pulmonary source of infection was assumed, which is a known phenomenon in domestic pigs [22].

### Maintenance and rewarming period

During maintenance deviation from pulmonary target temperature was 0.1±0.4°C (OHE11) and 0.0±0.1°C (OHE14.7, p = 0.91). Rewarming rates were 0.2±0.1°C/h (OHE11) and 0.2±0.1°C/h (OHE14.7; p = 0.75).

## Discussion

In the present study, OHE with diameters of 11 mm and 14.7 mm provided effective cooling rates during the experimental TTM-study-protocol. No clinically relevant oesophageal tissue damage was observed. Additionally, clinically relevant deviations from target temperature during maintenance were not found. Rewarming corresponded to an intended rewarming rate of 0.25°C/h.

### Cooling, maintenance and rewarming using OHE

To date, the optimal time to (pulmonary) target temperature after out-of-hospital cardiac arrest (OHCA) still remains unclear. Scientific evidence hints towards neuroprotection by rapid cooling after OHCA with 1–2°C/h [12]. Recently, Markota et al. reported a cooling rate of 1.12°C/h in a prospective evaluation of OHE after OHCA [23]. A cooling rate of 1.3°C/h was reached by OHE in an experimental study in pigs that did not suffer cardiac arrest but underwent a TTM-protocol [15]. Accordingly, cooling rates in the present study met the recommendations of rapid cooling with cooling rates between 2.8 and 3.0°C/h. However, we must not forget the fact that this was an experimental study with an average body weight of 27-30kg, which is approximately half of that used in the studies of Markota and Kulstad [15, 23]. Eventually, extrapolation of data of this study to experimental and clinical cooling rates is difficult, which is a limitation of this study. However, it can be concluded that diameters of OHE do not have a significant impact on the cooling rate in the present study, which could be of interest for further clinical use of OHE.

Furthermore, maintenance and rewarming rates of 0.25°C/h were accurate in the present study. To provide similar conditions during the rewarming period to the TTM-protocol used in our hospital, pigs were covered with a silver blanket known to decrease the incidence of shivering and passively increase the patient´s skin temperature [24]. However, accurate rewarming of approximately 0.25°C/h could also be confirmed experimentally by use of OHE in other studies [15–17, 23]. Interestingly, in two preceding studies in humans, preventing perioperative hypothermia with OHE was ineffective during hip [25] and abdominal surgery [26]. However, in both studies the operating area was large and could not be covered during surgery, which may enhance heat loss.

### Design of oesophageal heat exchanger

The extent of heat-convection between the body core and OHE may depend on the diameter of OHE. Although larger OHE diameters provide both higher flow rates within the tube and a larger exchange surface, the risk of pressure-related oesophageal tissue damage could also be increased. In search of an appropriate diameter of OHE, a broad spectrum of similar devices routinely placed in the oesophagus including gastric tubes (up to 11mm), an oesophageal echocardiography probe (14.7mm) and a Sengstaken-Blakemore tube (36mm) come into question. Eventually, the diameters of OHE11 and OHE14.7 used in the present study were based on diameters from lower and middle size of devices regarded as being safe [27]. Interestingly, an increase of the exchange surface from approximately 95m^2^ (OHE11) to approximately 147m^2^ (OHE14.7) did neither have substantial effects on oesophageal tissue damage nor on cooling-rates (Fig 2). Analogously, significant differences in energy gained by the patient (dt) were not found between OHE11 and OHE14.7. *dt* is a product of the flow rate within the OHE (*mass*/*time*[(*L*/min)]), constant heat capacity of water (4.186*kJ*/[*kg* * *K*]) and temperature of the cooling fluid (△T* = *T*_*in*_–*T*_*out*_). Due to a lower flow rate in OHE11, time provided for temperature convection is larger compared to OHE of larger diameters. Thus, Tout was significant higher in OHE11. In contrast, higher flow rates in OHE14.7 provide a higher exchange of the cooling mass (water). Accordingly, *dt* between both OHE was not different, which is also mirrored by the lack of difference in cooling rates. Possibly, other factors such as the pig´s blood flow, differences in body weight, mean ambient temperature may play a role in OHE-related cooling, but could not be included in our mathematical model.

### Histological damage related to OHE

It has been demonstrated that thermal tissue damage is inversely related to the degree of thermal energy and the extent of time [28, 29]. For instance, a temperature of 44°C for 6h was shown to cause irreversible damage at the basal cell level in the skin of guinea-pigs [30]. However, the constant tissue pressure applied by the OHE has also to be taken into account. In that respect, Kokate et al. found tissue damage in deeper layers at 35°C of temperature when a constant pressure of 100mmHg over a period of 5h was applied to the skin of pigs [31]. Given the tensile fold-structure of the oesophagus, the OHE-related pressure was assumed to be substantially lower than 100mmHg in the present study.

Another important point taken into consideration was the thermal threshold of 43°C known to initiate denaturation of proteins in general [30, 32]. Given a thermal dosage of 45°C x 30min [33] able to induce epithelial oesophageal damage [20, 34], an intraoesophageal upper temperature threshold of 41°C, which was the maximum temperature applied to the oesophagus by the OHE in the present study, seemed to be safe in minimizing the incidence of thermal oesophageal tissue damage [35]. Presumably, therefore, tissue damage was absent in cranial, middle and caudal oesphageal tissue segments. However, mild acute inflammatory transepithelial invasion was found in the laryngeal tissue segments of the oesophagus in all groups. The nearby cartilage structure of the larynx impedes relaxation of the oesophagus. Conversely, vertical folds enable the cranial, middle and caudal oesophageal tissue segments to distend to 2x3 cm during swallowing [36]. Therefore, one may assume that mild acute inflammation of laryngeal tissue segments is related to permanent high contact pressure of the OHE in the present study. Although all animals were treated identical prior and during the experiments tissue damage in laryngeal segments in the control group did not differ from tissue damage in laryngeal segments in the OHE11- and OHE14.7-group. It is important to mention that mild acute transepithelial invasion of inflammatory cells has also been found during physiological food impaction in pigs, which would also explain the high standard deviation in the laryngeal tissue segments [37]. Unfortunately, laryngeal segments were not examined in the first animals used. After accidentally observing an oedema during harvest in one animal the larynx were also removed and evaluated as well. Summarizing the histological results, oesophageal tissue damage should have been reversible in all animals and use of OHE does not seem to harm the oesophageal tissue.

### Limitations of the study

This experimental study was conducted in pigs that reveal a significant lower body weight compared to humans. However, evaluation of different cooling capabilities of OHE11 and OHE14.7 was of particular interest. Although the results of the present study need to be applied to clinical conditions with high caution, potential differences in cooling rates could have been shown in the present study. Moreover, the study protocol was adjusted to a maintenance of only one hour, which is significantly shorter compared to clinical conditions. However, duration of the cooling and rewarming period were comparable and our results are in line with clinical observations that did not find oesophageal tissue damage after treatment with OHE [16, 17] with comparable TTM-protocols (approximately 30h in lengths). To further asses long-term oesophageal tissue damage after use of OHE clinical and experimental studies with larger sample-sizes, cooling protocols >24h and follow-up examinations would be beneficial. In addition, the study was conducted in absence of OHCA. Core body temperature decline may be influenced by the perfusion of the tissue, preferably mirrored by the blood pressure that may be attenuated after OHCA.

The oesophageal heat exchanger (OHE) is intended to be inserted in comatose survivors of cardiac arrest (CA). These patients require anaesthesia after ROSC and admission to the ICU respectively. To date, there is a large body of evidence that general anaesthetics, such as fentanyl, propofol and midazolam degenerate vegetative thermoregulatory control. After induction of anaesthesia, core temperature can decrease up to 1°C in 30min due to a core-to-peripheral redistribution of body-heat. Thus, the decline of core body temperature may also be explained in part as a side-effect of anaesthetics [38, 39]. As reported, redistribution hypothermia could be impeded by warming patients before and immediately after induction of anaesthesia, respectively [38]. Thus, pigs were actively rewarmed during surgical preparation by covering with air-circulating blankets. Thereby, we reached a comparable baseline temperature in both groups prior to cooling and could also avoid inhomogeneous baseline temperatures. Given that thermoregulatory vasoconstriction is initiated at 34.5°C, which leads to relatively constant body temperatures between 33–34°C, an OHE-related effect can also be questioned during maintenance [38, 40]. However, we observed only small deviations from the target temperature of 33°C and therefore conclude that the OHE significantly influences cooling during this period.

## Conclusions

OHE with diameters of 11 mm and 14.7 mm achieve effective cooling rates for TTM and OHE did not cause any relevant oesophageal damage. Both OHE demonstrated acceptable deviations from target temperature and allowed for an intended rewarming rate (0.25°C/h).

## Supporting information

S1 Table Specific oesophageal tissue pathologies examined after treatment with OHE11, OHE14.7 or control animals. The sum was calculated for each slide.
(TIF)

## Abbreviations

IVD: Intravascular cooling device
OHCA: Out-of-hospital cardiac arrest
OHE: Oesophageal heat exchanger
TTM: Targeted temperature management

## References

[1] Jalan R, SW OD, Deutz NE, Lee A, Hayes PC. Moderate hypothermia for uncontrolled intracranial hypertension in acute liver failure. Lancet (London, England). 1999;354(9185):1164–8. Epub 1999/10/08. 10.1016/s0140-6736(98)12440-6 10513710

[2] Shankaran S, Laptook AR, Ehrenkranz RA, Tyson JE, McDonald SA, Donovan EF, et al Whole-body hypothermia for neonates with hypoxic-ischemic encephalopathy. The New England journal of medicine. 2005;353(15):1574–84. Epub 2005/10/14. 10.1056/NEJMcps050929 16221780

[3] Popugaev KA, Savin IA, Oshorov AV, Kurdumova NV, Ershova ON, Lubnin AU, et al Postsurgical meningitis complicated by severe refractory intracranial hypertension with limited treatment options: the role of mild therapeutic hypothermia. Journal of neurological surgery reports. 2014;75(2):e224–9. Epub 2014/12/09. PubMed Central PMCID: PMCPMC4242895. 10.1055/s-0034-1387188 25485219 PMC4242895

[4] Gotberg M, Olivecrona GK, Engblom H, Ugander M, van der Pals J, Heiberg E, et al Rapid short-duration hypothermia with cold saline and endovascular cooling before reperfusion reduces microvascular obstruction and myocardial infarct size. BMC cardiovascular disorders. 2008;8:7 Epub 2008/04/12. PubMed Central PMCID: PMCPMC2323360. 10.1186/1471-2261-8-7 18402663 PMC2323360

[5] Kleinman ME, Chameides L, Schexnayder SM, Samson RA, Hazinski MF, Atkins DL, et al Part 14: pediatric advanced life support: 2010 American Heart Association Guidelines for Cardiopulmonary Resuscitation and Emergency Cardiovascular Care. Circulation. 2010;122(18 Suppl 3):S876–908. Epub 2010/10/22. 20956230 10.1161/CIRCULATIONAHA.110.971101

[6] Saito T, Saito S, Yamamoto H, Tsuchida M. Neuroprotection following mild hypothermia after spinal cord ischemia in rats. Journal of vascular surgery. 2013;57(1):173–81. Epub 2012/11/28. 10.1016/j.jvs.2012.05.101 23182159

[7] Sydenham E, Roberts I, Alderson P. Hypothermia for traumatic head injury. The Cochrane database of systematic reviews. 2009;(2):Cd001048 Epub 2009/04/17. 10.1002/14651858.CD001048.pub4 19160187

[8] Guluma KZ, Hemmen TM, Olsen SE, Rapp KS, Lyden PD. A trial of therapeutic hypothermia via endovascular approach in awake patients with acute ischemic stroke: methodology. Academic emergency medicine: official journal of the Society for Academic Emergency Medicine. 2006;13(8):820–7. Epub 2006/06/13. 16766740 10.1197/j.aem.2006.03.559

[9] Schortgen F, Clabault K, Katsahian S, Devaquet J, Mercat A, Deye N, et al Fever control using external cooling in septic shock: a randomized controlled trial. American journal of respiratory and critical care medicine. 2012;185(10):1088–95. Epub 2012/03/01. 10.1164/rccm.201110-1820OC 22366046

[10] Nolan JP, Soar J, Cariou A, Cronberg T, Moulaert VR, Deakin CD, et al European Resuscitation Council and European Society of Intensive Care Medicine Guidelines for Post-resuscitation Care 2015: Section 5 of the European Resuscitation Council Guidelines for Resuscitation 2015. Resuscitation. 2015;95:202–22. Epub 2015/10/20. 10.1016/j.resuscitation.2015.07.018 26477702

[11] Hoedemaekers CW, Ezzahti M, Gerritsen A, van der Hoeven JG. Comparison of cooling methods to induce and maintain normo- and hypothermia in intensive care unit patients: a prospective intervention study. Critical care (London, England). 2007;11(4):R91. Epub 2007/08/28. PubMed Central PMCID: PMCPmc2206487. 10.1186/cc6104 PMC2206487 17718920

[12] Polderman KH, Herold I. Therapeutic hypothermia and controlled normothermia in the intensive care unit: practical considerations, side effects, and cooling methods. Critical care medicine. 2009;37(3):1101–20. Epub 2009/02/25. 10.1097/CCM.0b013e3181962ad5 19237924

[13] Lefrant JY, Muller L, de La Coussaye JE, Benbabaali M, Lebris C, Zeitoun N, et al Temperature measurement in intensive care patients: comparison of urinary bladder, oesophageal, rectal, axillary, and inguinal methods versus pulmonary artery core method. Intensive care medicine. 2003;29(3):414–8. Epub 2003/02/11. 10.1007/s00134-002-1619-5 12577157

[14] Kulstad EB, Courtney DM, Waller D. Induction of therapeutic hypothermia via the esophagus: a proof of concept study. World journal of emergency medicine. 2012;3(2):118–22. Epub 2012/01/01. PubMed Central PMCID: PMCPMC4129793. 10.5847/wjem.j.1920-8642.2012.02.007 25215049 PMC4129793

[15] Kulstad E, Metzger AK, Courtney DM, Rees J, Shanley P, Matsuura T, et al Induction, maintenance, and reversal of therapeutic hypothermia with an esophageal heat transfer device. Resuscitation. 2013;84(11):1619–24. Epub 2013/07/06. 10.1016/j.resuscitation.2013.06.019 23827887

[16] Markota A, Kosir AS, Balazic P, Zivko I, Sinkovic A. A Novel Esophageal Heat Transfer Device for Temperature Management in an Adult Patient with Severe Meningitis. The Journal of emergency medicine. 2016. Epub 2016/09/24. 10.1016/j.jemermed.2016.07.086 27658559

[17] Markota A, Kit B, Fluher J, Sinkovic A. Use of an oesophageal heat transfer device in therapeutic hypothermia. Resuscitation. 2015;89:e1–2. Epub 2015/02/11. 10.1016/j.resuscitation.2015.01.032 25660954

[18] Kilkenny C, Browne WJ, Cuthill IC, Emerson M, Altman DG. Improving bioscience research reporting: the ARRIVE guidelines for reporting animal research. PLoS biology. 2010;8(6):e1000412 Epub 2010/07/09. PubMed Central PMCID: PMCPmc2893951. 10.1371/journal.pbio.1000412 20613859 PMC2893951

[19] Pehbock D, Dietrich H, Klima G, Paal P, Lindner KH, Wenzel V. Anesthesia in swine: Optimizing a laboratory model to optimize translational research. Der Anaesthesist. 2014. Epub 2014/11/12. 10.1007/s00101-014-2371-2 25384955

[20] Lequerica JL, Sanz E, Hornero F, Herrero M, Ruiz N, Burdio F, et al Esophagus histological analysis after hyperthermia-induced injury: implications for cardiac ablation. International journal of hyperthermia: the official journal of European Society for Hyperthermic Oncology, North American Hyperthermia Group. 2009;25(2):150–9. Epub 2009/04/02. 10.1080/02656730802537626 19337915

[21] Naiman M, Shanley P, Garrett F, Kulstad E. Evaluation of advanced cooling therapy's esophageal cooling device for core temperature control. Expert review of medical devices. 2016;13(5):423–33. Epub 2016/04/05. 10.1080/17434440.2016.1174573 27043177

[22] Ran Z, Shen H, Lang Y, Kolb EA, Turan N, Zhu L, et al Domestic pigs are susceptible to infection with influenza B viruses. Journal of virology. 2015;89(9):4818–26. Epub 2015/02/13. PubMed Central PMCID: PMCPMC4403465. 10.1128/JVI.00059-15 25673727 PMC4403465

[23] Markota A, Fluher J, Kit B, Balazic P, Sinkovic A. The introduction of an esophageal heat transfer device into a therapeutic hypothermia protocol: A prospective evaluation. The American journal of emergency medicine. 2016;34(4):741–5. Epub 2016/02/26. 10.1016/j.ajem.2016.01.028 26906333

[24] Buggy D, Hughes N. Pre-emptive use of the space blanket reduces shivering after general anaesthesia. British journal of anaesthesia. 1994;72(4):393–6. Epub 1994/04/01. 8155437 10.1093/bja/72.4.393

[25] Kulkarni P, Matson A, Bright J, Pearson J, Carli F. Clinical evaluation of the oesophageal heat exchanger in the prevention of perioperative hypothermia. British journal of anaesthesia. 1993;70(2):216–8. Epub 1993/02/01. 8435268 10.1093/bja/70.2.216

[26] Rasmussen YH, Leikersfeldt G, Drenck NE. Forced-air surface warming versus oesophageal heat exchanger in the prevention of peroperative hypothermia. Acta anaesthesiologica Scandinavica. 1998;42(3):348–52. Epub 1998/05/23. 9542564 10.1111/j.1399-6576.1998.tb04928.x

[27] Piercy M, McNicol L, Dinh DT, Story DA, Smith JA. Major complications related to the use of transesophageal echocardiography in cardiac surgery. Journal of cardiothoracic and vascular anesthesia. 2009;23(1):62–5. Epub 2008/12/09. 10.1053/j.jvca.2008.09.014 19058977

[28] Hudack S, McMaster PD. THE GRADIENT OF PERMEABILITY OF THE SKIN VESSELS AS INFLUENCED BY HEAT, COLD, AND LIGHT. The Journal of experimental medicine. 1932;55(3):431–9. Epub 1932/02/29. PubMed Central PMCID: PMCPMC2132116. 19870001 10.1084/jem.55.3.431 PMC2132116

[29] McMaster PD, Hudack SS. THE PARTICIPATION OF SKIN LYMPHATICS IN REPAIR OF THE LESIONS DUE TO INCISIONS AND BURNS. The Journal of experimental medicine. 1934;60(4):479–501. Epub 1934/09/30. PubMed Central PMCID: PMCPMC2132402. 19870317 10.1084/jem.60.4.479 PMC2132402

[30] Henriques FC, Moritz AR. Studies of Thermal Injury: I. The Conduction of Heat to and through Skin and the Temperatures Attained Therein. A Theoretical and an Experimental Investigation. The American journal of pathology. 1947;23(4):530–49. Epub 1947/07/01. PubMed Central PMCID: PMCPMC1934298. 19970945 PMC1934298

[31] Kokate JY, Leland KJ, Held AM, Hansen GL, Kveen GL, Johnson BA, et al Temperature-modulated pressure ulcers: a porcine model. Archives of physical medicine and rehabilitation. 1995;76(7):666–73. Epub 1995/07/01. 7605187 10.1016/s0003-9993(95)80637-7

[32] He X, Bischof JC. Quantification of temperature and injury response in thermal therapy and cryosurgery. Critical reviews in biomedical engineering. 2003;31(5–6):355–422. Epub 2004/05/14. 15139301 10.1615/critrevbiomedeng.v31.i56.10

[33] Li DJ, Zhou SL, Qiu SL, Qiao SJ. Thermodamage, thermosensitivity and thermotolerance of normal swine oesophagus. International journal of hyperthermia: the official journal of European Society for Hyperthermic Oncology, North American Hyperthermia Group. 1987;3(2):143–51. Epub 1987/03/01. 10.3109/02656738709140382 3598250

[34] Tobey NA, Sikka D, Marten E, Caymaz-Bor C, Hosseini SS, Orlando RC. Effect of heat stress on rabbit esophageal epithelium. The American journal of physiology. 1999;276(6 Pt 1):G1322–30. Epub 1999/06/11. 10362635 10.1152/ajpgi.1999.276.6.G1322

[35] Sause A, Tutdibi O, Pomsel K, Dinh W, Futh R, Lankisch M, et al Limiting esophageal temperature in radiofrequency ablation of left atrial tachyarrhythmias results in low incidence of thermal esophageal lesions. BMC cardiovascular disorders. 2010;10:52 Epub 2010/10/28. PubMed Central PMCID: PMCPmc2987899. 10.1186/1471-2261-10-52 20977747 PMC2987899

[36] Long J, Orlando R. Anatomy, histology, embryology, and developmental abnormalities of the esophagus Gastrointestinal and Liver Diseases. WB Saunders: Feldman M Fieldman LS Sleisenger MH; 2002 p. 551–60.

[37] Desai TK, Stecevic V, Chang CH, Goldstein NS, Badizadegan K, Furuta GT. Association of eosinophilic inflammation with esophageal food impaction in adults. Gastrointestinal endoscopy. 2005;61(7):795–801. Epub 2005/06/04. 15933677 10.1016/s0016-5107(05)00313-5

[38] Sessler DI. The thermoregulation story. Anesthesiology. 2013;118(1):181–6. Epub 2012/12/12. 10.1097/ALN.0b013e3182784df3 23221865

[39] Sessler DI, Olofsson CI, Rubinstein EH, Beebe JJ. The thermoregulatory threshold in humans during halothane anesthesia. Anesthesiology. 1988;68(6):836–42. Epub 1988/06/01. 3377230 10.1097/00000542-198806000-00002

[40] Sessler DI. Temperature monitoring and perioperative thermoregulation. Anesthesiology. 2008;109(2):318–38. Epub 2008/07/24. PubMed Central PMCID: PMCPmc2614355. 10.1097/ALN.0b013e31817f6d76 18648241 PMC2614355

