# Supplementary figures and images for: Oesophageal heat exchangers with a diameter of 11mm or 14.7mm are equally effective and safe for targeted temperature management
Source: PLoS One. 2017 Mar 14;12(3):e0173229. doi: 10.1371/journal.pone.0173229 (PMC5349448; doi:10.1371/journal.pone.0173229)

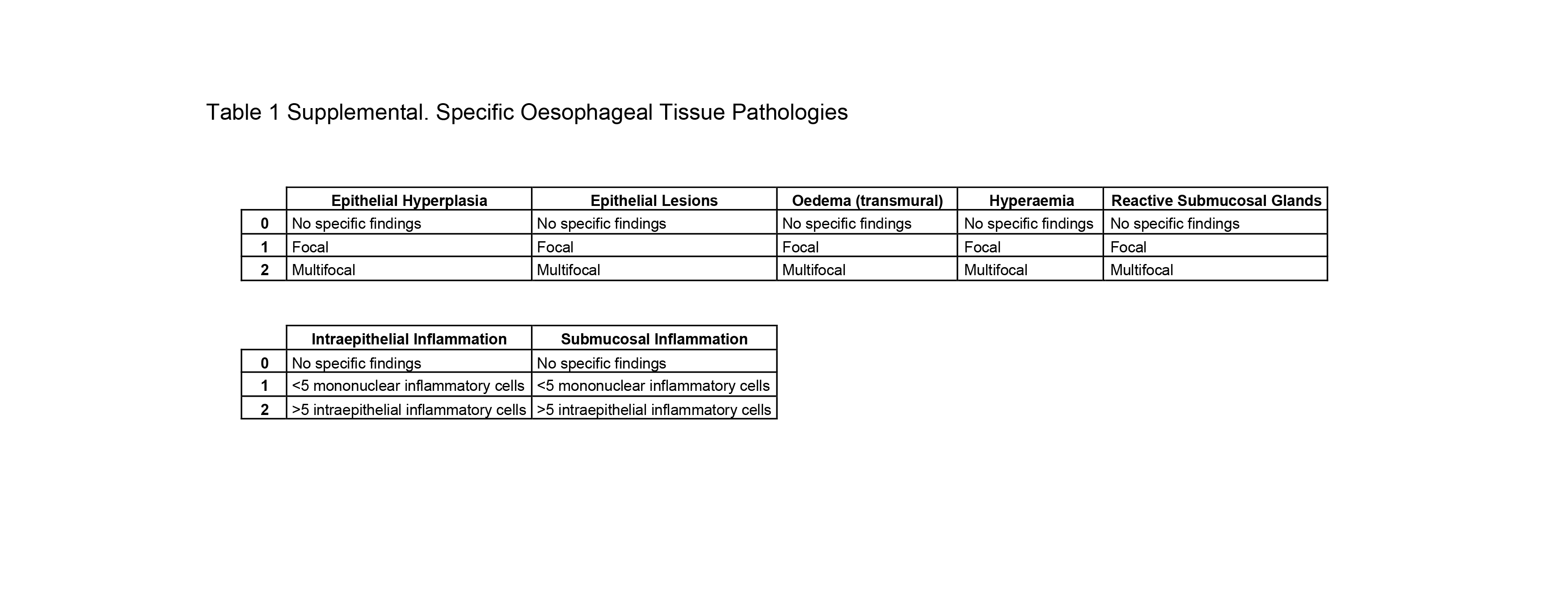

Supplement: S1 Table — The sum was calculated for each slide. (TIF) [file pone.0173229.s001.TIF]
